# Taiwanese consumer survey data for investigating the role of information on equivalence of organic standards in directing food choice

**DOI:** 10.1016/j.dib.2018.03.054

**Published:** 2018-03-17

**Authors:** Ching-Hua Yeh, Monika Hartmann, Stefan Hirsch

**Affiliations:** a Institute for Food and Resource Economics, University of Bonn, Germany; b AECP Group, ETH Zürich, Switzerland

## Abstract

The presentation of credence attributes such as the product's origin or the production method has a significant influence on consumers' food purchase decisions. The dataset includes survey responses from a discrete choice experiment with 1309 food shoppers in Taiwan using the example of sweet pepper. The survey was carried out in 2014 in the three largest Taiwanese cities. It evaluates the impact of providing information on the equality of organic standards on consumers' preferences at the example of sweet pepper. Equality of organic standards implies that regardless of products’ country-of-origin (COO) organic certifications are based on the same production regulation and managerial processes. Respondents were randomly allocated to the information treatment and the control group. The dataset contains the product choices of participants in both groups, as well as their sociodemographic information.

**Specifications Table**

**Table:** 

Subject area	*Experimental economics*
More specific subject area	*Discrete choice experiment (DCE)*
Type of data	*Excel tables, CSV file*
How data was acquired	*Personal interviews combined with internet-connected electronic devices*
Data format	*Raw*
Experimental factors	*Treatment and control group where the former was provided with information regarding the equality of organic standards independent of country-of-origin*
Experimental features	*Country-of-origin, production methods, chemical residue testing and price as well as information treatment*
Data source location	*Taipei city/Taichung city/ Kaohsiung city in Taiwan*
Data accessibility	*With this article*

**Value of the data**

• This data set provides a first-hand survey that explores the relevance of process attributes for consumers' food choice in the context of an emerging nation.
• The data can be used for comparing the importance of labels related to process attributes with other case studies in a meta-analysis.
• The data allows for a comparison of the effects of information treatment regarding food process labels on the importance of those labels in consumers’ choice in a meta-analysis.
• The data allows to explore the influence of alternative statistical approaches in estimating consumers' preference scores for various process attributes.

## 1 Data

The dataset contains raw data collected in 2014 from 1309 Taiwanese consumers by combining personal interviews with internet connected electronic devices. The survey was carried out in the three largest Taiwanese cities: Kaohsiung city, Taichung city, New Taipei city^1^. The analysis focuses on sweet pepper, as this product is part of Taiwanese daily diet and available in conventional and organic quality from domestic producers as well as from third countries. The questionnaire is designed to assess the impact information on the equality of organic standards has on the importance consumers attach to the attributes COO, production method, chemical residual testing and price using a choice experimental design. Additionally, the data includes 13 individual-specific sociodemographic variables. The data was first analyzed in the article by Yeh et al. [1].

## 2 Experimental design, materials and methods

A random sampling approach for the selection of participants and their assignment to the treatment and control group was conducted to ensure that both samples are representative of food shoppers in urban Taiwanese regions. The questionnaire started with two screening questions. The screening questions allow filtering the relevant participants for those i) responsible for their households’ food shopping, and ii) regularly consuming sweet peppers. 509 respondents did not fulfill one or both of those criteria resulting in 469 consumers in the control and 331 consumers in the treatment group.

The choice experiment focused on the following attributes and attribute levels of sweet pepper that were derived from focus group discussions and identified as most important^2^: (i) *Country-of-origin* with the attribute levels, Taiwan, China and Japan, (ii) *Production method* with attribute levels organic and conventional, (iii) *Chemical residue testing* comprising three attribute levels: Chemical residual testing approved in the production country, respective testing approved in Taiwan, and no chemical residual information, and (iv) *Price* with the levels 65 NT^3^, 85 NT, 105 NT, and 125 NT.

The DCE experimental design consists of 36 choice sets each comprising three alternatives. The 36 choice sets are allocated across six blocks and participants were randomly allocated to one of these blocks. The supplementary table entitled DCE experimental design provides an overview of the distribution of choice sets and alternatives across the six blocking versions. In the dataset information on the alternative chosen in each choice set is captured by “DCE1” to “DCE6” while information on the blocking version each participant was assigned to is provided in the column “DCE blocking version”.

Prior to the discrete choice experiment participants in the information treatment group obtained the following information on the equality of organic standards:

“*No matter where an organic product sold in Taiwan has been produced, the same regulation and managerial processes apply. A product that is labeled organic and sold in Taiwan has to fulfill the Taiwanese organic production regulations and it is ensured that there are no exceptions.*”

Moreover, 13 individual-specific covariates that potentially have an impact on the DCE outcome as well as on the information treatment were included [2], [3]: gender, age range, living location (i.e. in the north/ middle/ south of Taiwan), living area (i.e. in a big city/ mid-sized city/ rural area), education, marital status, career, household size, number of children, monthly household net income, monthly household food expenses, the average share of imported food in total household and the blocking version of discrete choice experiment.

## Funding

This research did not receive any specific grant from funding agencies in the public, commercial, or not-for-profit sectors.

## Appendix A Supplementary material

Supplementary material The choice experimental design, an example of a choice task as well as well as the variable descriptions are included in the Supplementary file.

## Footnotes

1 New Taipei city is located in the north, and the largest city in Taiwan. Taichung city is located in the central area and the third most populated area in Taiwan. Kaohsiung city is the largest city in the south, and the second largest city in Taiwan.

2 The focus group discussions were held via video meetings with Taiwanese food market experts in March, 2014.

3 New Taiwanese dollars. In July 2014, 1 US Dollar=29.98 NT.
