# Supplementary material for: Taiwanese consumer survey data for investigating the role of information on equivalence of organic standards in directing food choice
Source: Data Brief. 2018 Mar 17;18:688–90. doi: 10.1016/j.dib.2018.03.054 (PMC5996284; doi:10.1016/j.dib.2018.03.054)
Supplement: Supplementary file 1 — Supplementary material [file mmc3.docx]

**Conflict of interest and author declaration form**

**Title of article:** Taiwanese consumer survey data for investigating the role of information on equivalence of organic standards in directing food choice

**Authors:** Ching-Hua Yeh^a^, Monika Hartmann^a^, Stefan Hirsch^b^

**Affiliations:** *^a^Institute for Food and Resource Economics, University of Bonn, Germany*

*^b^AECP Group, ETH Zürich, Switzerland*

**Contact email:** chinghua.yeh@ilr.uni-bonn.de

**Declaration:** This statement is to clarify that all authors have approved the manuscript being submitted to Data in Brief. The authors of this article have not received any funding and have no conflict of interest to declare.
