# Supplementary material for: Taiwanese consumer survey data for investigating the role of information on equivalence of organic standards in directing food choice
Source: Data Brief. 2018 Mar 17;18:688–90. doi: 10.1016/j.dib.2018.03.054 (PMC5996284; doi:10.1016/j.dib.2018.03.054)
Supplement: Supplementary file 2 — Supplementary material The choice experimental design, an example of a choice task as well as well as the variable descriptions are included in the Supplementary file. [file mmc1.pdf]

DCE experimental design

Version	Choice set	Alternative	Att 1 - Country of origin	Att 2 - Production method	Att 3 - Chemical residue testing	Att 4 - Price
1	1	1	2	1	1	2
1	1	2	2	1	2	2
1	1	3	1	2	3	1
1	2	1	1	2	2	2
1	2	2	2	1	1	1
1	2	3	3	1	2	2
1	3	1	2	1	1	2
1	3	2	1	2	3	1
1	3	3	3	2	3	4
1	4	1	3	2	3	4
1	4	2	1	2	2	2
1	4	3	2	1	1	1
1	5	1	2	2	1	1
1	5	2	3	2	3	4
1	5	3	1	1	2	3
1	6	1	3	1	1	3
1	6	2	3	1	3	1
1	6	3	3	2	2	2
2	1	1	2	2	3	2
2	1	2	2	2	1	3
2	1	3	1	1	2	4
2	2	1	3	2	1	3
2	2	2	1	1	1	3
2	2	3	2	2	2	1
2	3	1	2	2	3	3
2	3	2	3	1	2	2
2	3	3	3	1	1	1
2	4	1	1	1	3	3
2	4	2	1	2	3	3
2	4	3	2	2	1	3
2	5	1	3	1	2	1
2	5	2	3	2	1	2
2	5	3	1	2	3	4
2	6	1	1	1	3	4
2	6	2	2	2	1	3
2	6	3	2	1	2	4
3	1	1	3	2	1	1
3	1	2	1	2	3	4
3	1	3	2	1	3	3

3	2	1	2	2	2	1
3	2	2	1	1	1	3
3	2	3	3	2	3	3
3	3	1	1	1	3	2
3	3	2	3	2	3	4
3	3	3	2	2	2	1
3	4	1	3	1	2	1
3	4	2	3	1	1	2
3	4	3	1	2	3	4
3	5	1	2	2	2	3
3	5	2	1	1	1	4
3	5	3	2	1	3	3
3	6	1	1	1	2	4
3	6	2	2	1	3	1
3	6	3	1	2	1	3
4	1	1	1	1	1	4
4	1	2	2	2	2	3
4	1	3	2	2	3	2
4	2	1	3	2	3	1
4	2	2	3	1	2	3
4	2	3	3	1	1	2
4	3	1	2	2	1	2
4	3	2	1	1	2	4
4	3	3	1	1	3	3
4	4	1	1	2	2	4
4	4	2	2	1	3	3
4	4	3	3	1	1	1
4	5	1	2	2	3	4
4	5	2	3	2	1	2
4	5	3	3	1	2	3
4	6	1	2	1	3	2
4	6	2	1	2	2	4
4	6	3	1	2	1	4
5	1	1	1	1	1	3
5	1	2	1	1	2	4
5	1	3	1	2	3	2
5	2	1	1	1	2	4
5	2	2	2	2	3	2
5	2	3	3	1	1	1
5	3	1	2	2	3	3
5	3	2	3	1	1	1
5	3	3	3	1	2	2
5	4	1	2	1	2	3
5	4	2	3	1	2	1

5	4	3	1	2	1	4
5	5	1	1	1	3	2
5	5	2	2	2	2	1
5	5	3	1	2	1	3
5	6	1	1	1	1	4
5	6	2	2	1	3	2
5	6	3	1	2	2	3
6	1	1	3	2	2	2
6	1	2	3	2	1	1
6	1	3	2	1	3	4
6	2	1	3	2	2	4
6	2	2	1	2	3	1
6	2	3	2	1	1	2
6	3	1	1	2	1	3
6	3	2	2	1	2	2
6	3	3	2	2	3	1
6	4	1	3	2	3	1
6	4	2	2	2	1	4
6	4	3	3	1	2	1
6	5	1	3	1	1	1
6	5	2	3	1	2	3
6	5	3	2	2	2	4
6	6	1	3	1	2	1
6	6	2	1	2	3	4
6	6	3	3	1	1	2
