# Supplementary material for: Taiwanese consumer survey data for investigating the role of information on equivalence of organic standards in directing food choice
Source: Data Brief. 2018 Mar 17;18:688–90. doi: 10.1016/j.dib.2018.03.054 (PMC5996284; doi:10.1016/j.dib.2018.03.054)
Supplement: Supplementary file 3 — Supplementary material [file mmc2.pdf]

Example of the DCE choice question

Original version in Mandarin

台灣消費者之購買選擇行為研究

接下來，您將會面臨六個甜椒採購的問題。在開始之前，我們想提醒您：

<u>所有在台灣市場上所販賣的有機食品，不論是由哪一國生產，均符合相同的有機食品法規與管理過程。</u>
<u>食品能被透過有機認證標章販賣，即代表了它符合了有機生產的要求。</u>

Note: This page is only shown to the (EqualOrganic) information treatment group

English translated version

In the next section, you will face 6 questions regarding fresh sweet peppers purchase. We would like to kindly remind you that:

“No matter where an organic product sold in Taiwan has been produced, the same regulation and managerial processes apply. A product that is labeled organic and sold in Taiwan has to fulfill the Taiwanese organic production regulations and it is ensured that there are no exceptions.”

台灣消費者之購買選擇行為研究

接下來您會面臨六個採購甜椒的問題。 請您想像自己正處於平時會去購物的那間超市內，您正準備採買紅甜椒。

假設所有紅甜椒的大小，顏色，成熟度，氣味等都是完全相同，但將以四種不同的產品資訊（產地、生產方式、是否通過農藥殘留檢驗及價格）提供您進行購買選擇。請您選出您最想購買的紅甜椒產品。

當然，您也可以自由選擇不想購買任何產品。不過，請注意，您所花費在購買紅甜椒的支出將會減少您能購買其它產品的預算！

請點擊下方箭頭開始作答。祝您購物愉快！

Please imagine that you are doing grocery shopping in a store that you usually go.

Below you will face 6 shopping tasks and will see the products' information (regarding origin, production methods, results of chemical residue testing and price) presented in these displays; simultaneously we assume that the red sweet peppers are identical in terms of size, color, ripeness, smell, etc.

Of course, you are free to make a decision of not buying any product, and please note that any purchase that you make will reduce the amount of money that you and your family have available for other purchases.

Please continue the survey by clicking the arrow button. Happy shopping!

以下三種甜椒，請問您會購買哪一種？

(1 of 6)

產品編號	1	2	3	4
	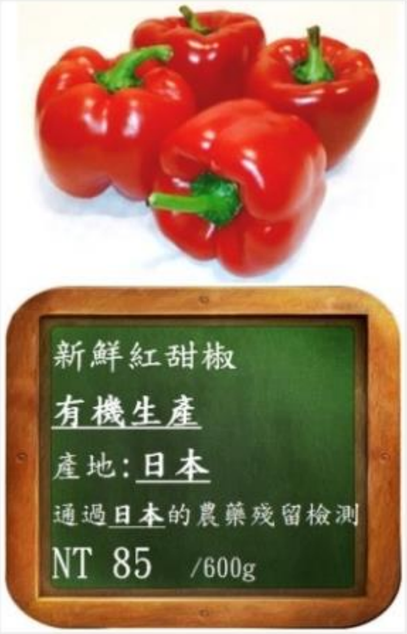	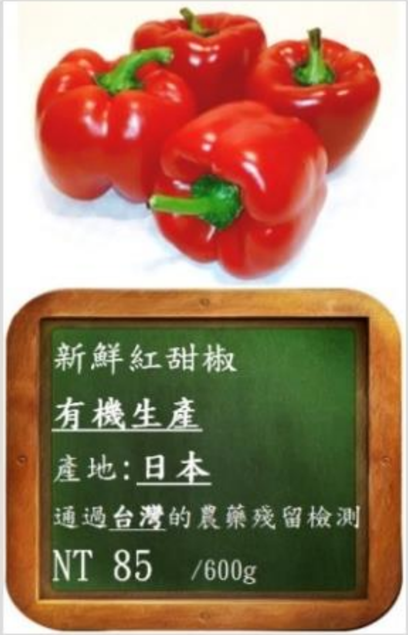	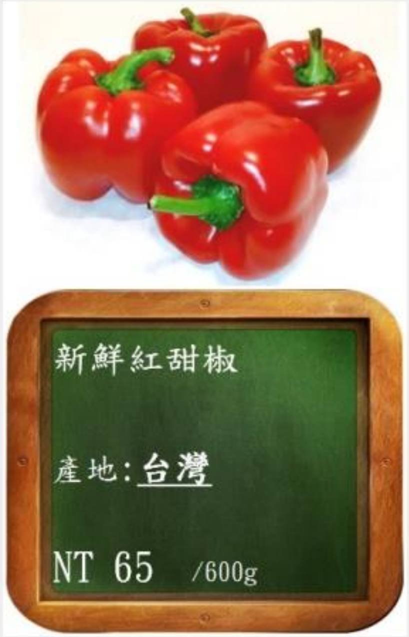	我不會購買這些甜椒。
請問您會購買哪一種甜椒？	○	○	○	○

請點擊箭頭圖案繼續，謝謝。

The following sweet peppers are available. Which one would you buy?

台灣消費者之購買選擇行為研究

以下三種甜椒，請問您會購買哪一種？

(2 of 6)

<table>
<tr><th>產品編號</th><th>1</th><th>2</th><th>3</th><th>4</th></tr>
<tr><td></td>
<td>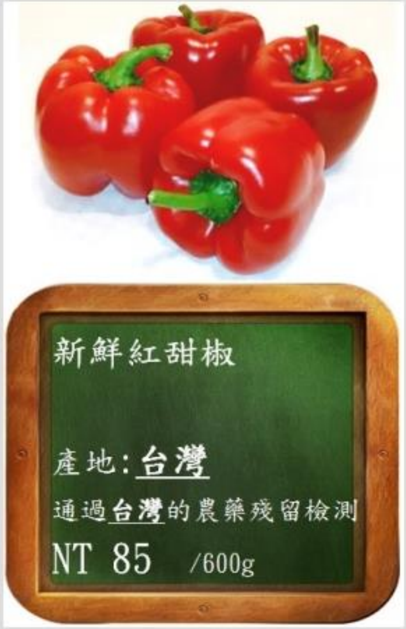
</td>
<td>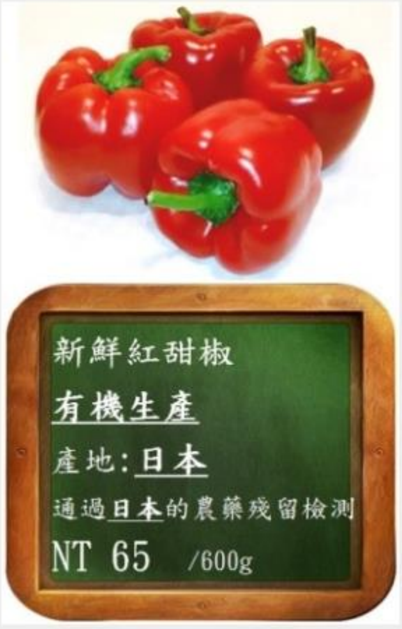
</td>
<td>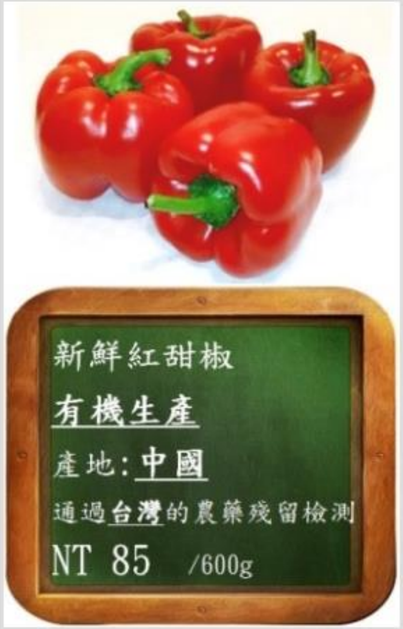
</td>
<td>我不會購買這些甜椒。</td></tr>
<tr><td>請問您會購買哪一種甜椒？</td><td>○</td><td>○</td><td>○</td><td>○</td></tr>
</table>

請點擊箭頭圖案繼續，謝謝。

◀ ▶

台灣消費者之購買選擇行為研究

以下三種甜椒，請問您會購買哪一種？

(3 of 6)

<table>
<tr><th>產品編號</th><th>1</th><th>2</th><th>3</th><th>4</th></tr>
<tr><td></td><td>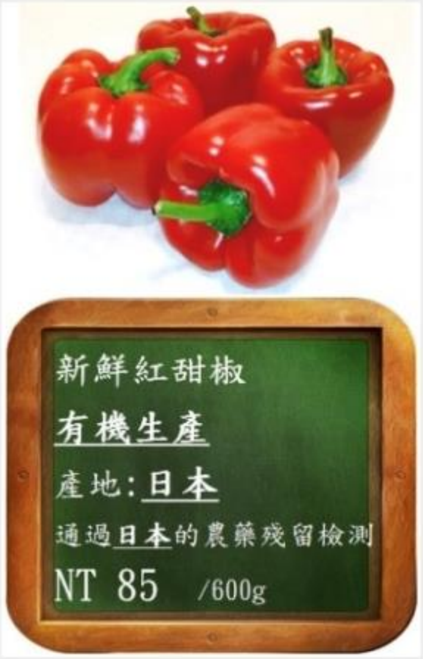
</td><td>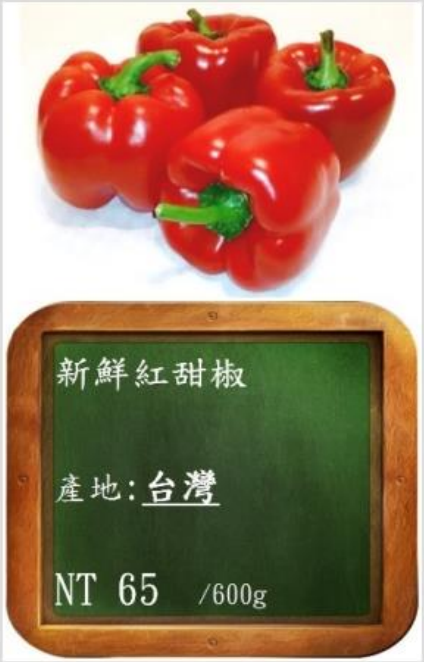
</td><td>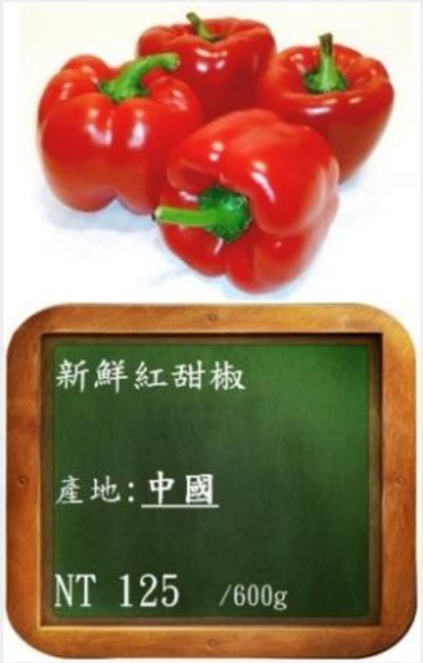
</td><td>我不會購買這些甜椒。</td></tr>
<tr><td>請問您會購買哪一種甜椒？</td><td>○</td><td>○</td><td>○</td><td>○</td></tr>
</table>

請點擊箭頭圖案繼續，謝謝。

以下三種甜椒，請問您會購買哪一種？

(4 of 6)

<table>
<tr><th>產品編號</th><th>1</th><th>2</th><th>3</th><th>4</th></tr>
<tr><td></td>
<td>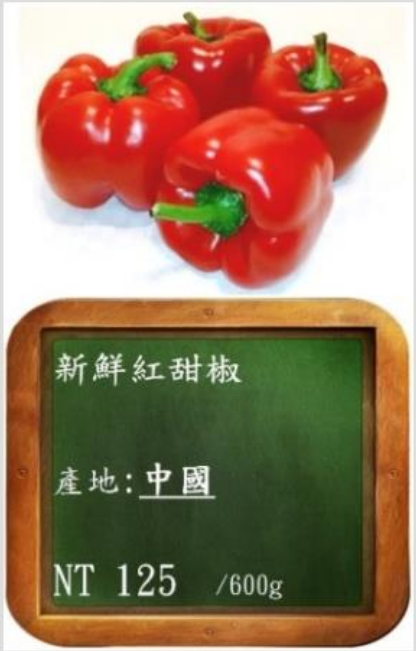
</td>
<td>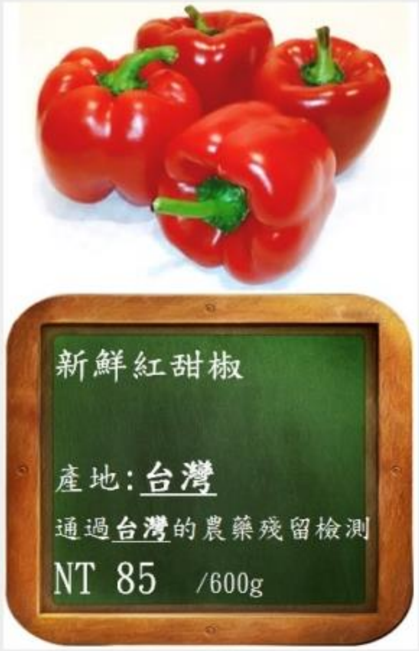
</td>
<td>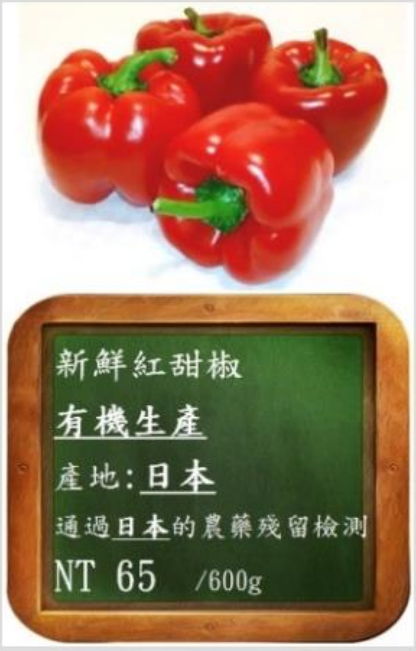
</td>
<td>我不會購買這些甜椒。</td></tr>
<tr><td>請問您會購買哪一種甜椒？</td><td>○</td><td>○</td><td>○</td><td>○</td></tr>
</table>

請點擊箭頭圖案繼續，謝謝。

◀ ▶

台灣消費者之購買選擇行為研究

以下三種甜椒，請問您會購買哪一種？

(5 of 6)

產品編號	1	2	3	4
	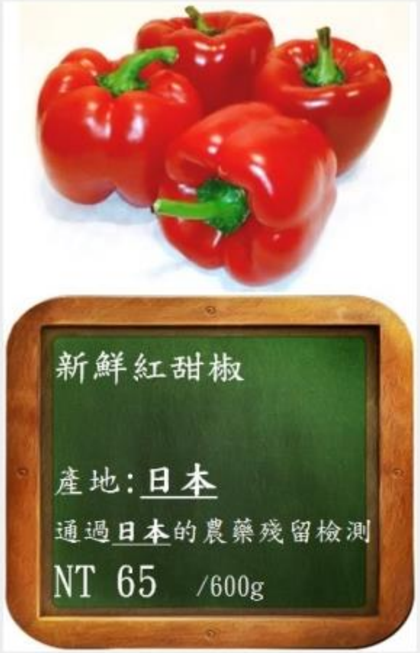	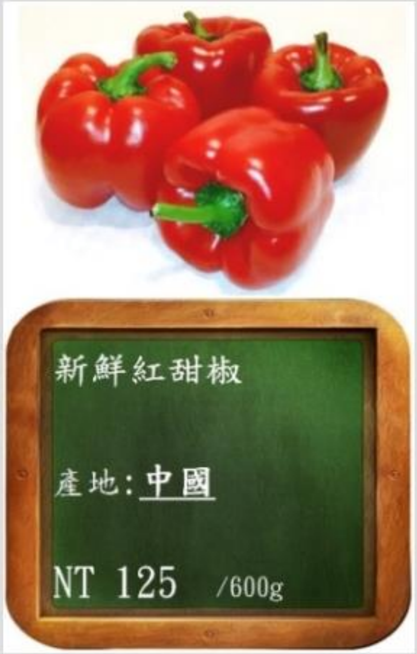	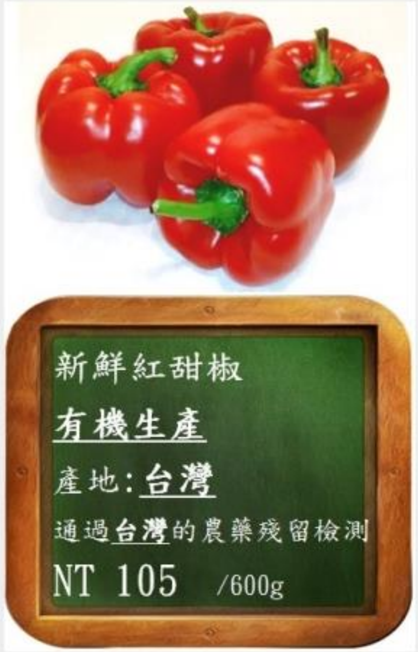	我不會購買這些甜椒。
**請問您會購買哪一種甜椒？**	○	○	○	○

請點擊箭頭圖案繼續，謝謝。

以下三種甜椒，請問您會購買哪一種？

(6 of 6)

產品編號	1	2	3	4
	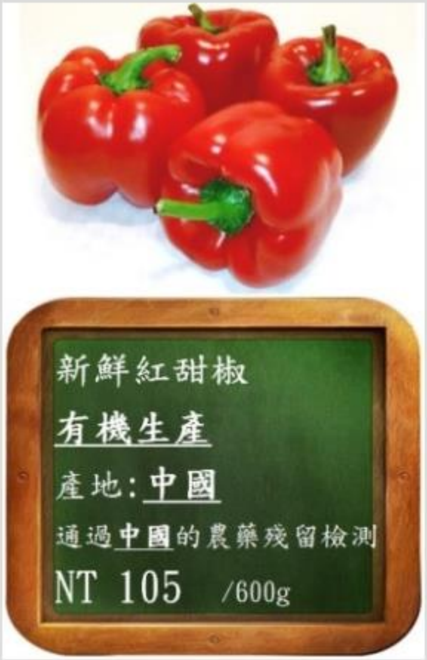	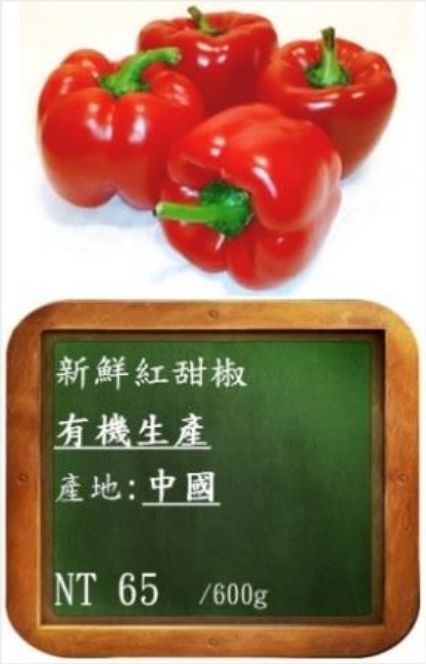	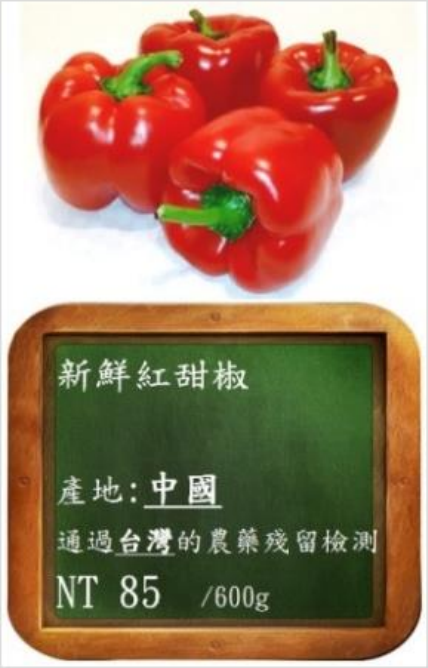	我不會購買這些甜椒。
請問您會購買哪一種甜椒？	○	○	○	○

請點擊箭頭圖案繼續，謝謝。
