# Supplementary material for: Taiwanese consumer survey data for investigating the role of information on equivalence of organic standards in directing food choice
Source: Data Brief. 2018 Mar 17;18:688–90. doi: 10.1016/j.dib.2018.03.054 (PMC5996284; doi:10.1016/j.dib.2018.03.054)
Supplement: Supplementary file 5 — Supplementary material [file mmc5.pdf]

Variable description:

ID	Respondents' ID number
Group	0 = Control group<br>1 = Information treatment group
ShoppingResponsibility	To what extent are you responsible for buying food in your household?<br>1 = Mainly responsible for household food shopping;<br>2 = Partly responsible for household food shopping;<br>3 = Not responsible for household food shopping
EatingPeppers	How do you consume sweet peppers?<br>1 = I eat it fresh directly;<br>2 = I eat it crashed in a juice form;<br>3 = I eat it warmly cooked;<br>4 = I eat it cured; 5= Others;<br>6 = I DO NOT eat sweet pepper
DCE1	The value indicates the number of the alternative was chosen in the choice task.
DCE2	The value indicates the number of the alternative was chosen in the choice task.
DCE3	The value indicates the number of the alternative was chosen in the choice task.
DCE4	The value indicates the number of the alternative was chosen in the choice task.
DCE5	The value indicates the number of the alternative was chosen in the choice task.
DCE6	The value indicates the number of the alternative was chosen in the choice task.
Gender	Your gender.<br>1 = Female;<br>2 = male
AgeRange	Your age range.<br>1 = 19 and under;<br>2 = 20-29;<br>3 = 30-39;<br>4 = 40-49;<br>5 = 50-59;<br>6 = 60 and over;<br>7 = No comment
LivingLocation	Please indicate the location where you live.<br>1 = Northern part of TW;<br>2 = Middle part of TW;<br>3 = Southern part of TW;<br>4 = Eastern part of TW
LivingArea	Please indicate which area you are living in Taiwan.<br>1 = Urban and large city area;<br>2 = Medium size city area;<br>3 = Rural and small city area
HouseholdSize	How many people are there in your household, including yourself?
KindsNumber	Of them, how many children (≤18 years old) currently live in your household?

MaritalStatus	Please indicate your marital status.<br>1 = Single;<br>2 = Single but in a relationship;<br>3 = Married;<br>4 = Others (i.e. Divorced, widowed etc.);<br>5 = No comment
Education	Please indicate the highest level of education you have achieved. 1 = Up to secondary school (9-year-education);<br>2 = High school (12 year education);<br>3 = College;<br>4 = University and higher;<br>5 = No comment
Career	Your career.<br>1 = Governmental servant;<br>2 = Employed in private company;<br>3 = Self-employed;<br>4 = Student;<br>5 = Housemaker;<br>6 = Retirement;<br>7 = Job hunting
HHNetIncome	Please indicate your household monthly net income range.<br>1 = Up to NT 20,000;<br>2 = NT 20,001 –NT 40,000;<br>3 = NT 40,001 –NT 60,000;<br>4 = NT 60,001 –NT 80,000;<br>5 = NT 80,001 –NT 100,000;<br>6 = NT 100,001– NT 120,000;<br>7 = Over NT 120,000;<br>8 = No comment
HHFoodExpenses	Please indicate the monthly food expenditure in your household.<br>1 = Up to NT 8,000;<br>2 = NT 8,001 – NT 16,000;<br>3 = NT 16,001– NT 24,000;<br>4 = NT 24,001 – NT 32,000;<br>5 = Over NT 32,001
ImportsBuyingProportion	Please indicate the average proportion of imported food in each grocery shopping.<br>1 = Up to 20%;<br>2 = 21%-40%;<br>3 = 41%-60%;<br>4 = 61%-80%;<br>5 = Over 80%;<br>6 = Never bought
DCE blocking version	The number of the blocking version
